# Malaysians' Preferences and Concerns Regarding Seeking Information About Illegal Drugs

**DOI:** 10.3389/fpubh.2018.00143

**Published:** 2018-05-17

**Authors:** Qiu Ting Chie, Cai Lian Tam, Gregory Bonn

**Affiliations:** ^1^Psychology, Universiti Tunku Abdul Rahman, Petaling Jaya, Malaysia; ^2^Psychology, Monash University Malaysia, Jeffrey Cheah School of Medicine and Health Sciences, Bandar Sunway, Malaysia; ^3^Psychology, General Studies, King Fahd University of Petroleum and Minerals, Dhahran, Saudi Arabia

**Keywords:** drug use, Malaysia, information seeking behavior, drug education, drug information

## Abstract

A brief survey asked Malaysians if they had searched for information about illegal drugs and their thoughts about the information available. Two hundred and eighty participants from four states: Selangor, Penang, Malacca, and Johor filled out a paper-and-pencil survey including both multiple choice and open-ended written questions. Quantitative analyses of closed-ended, and thematic analyses of open-ended data indicated the following: Half of participants had, at some point, actively searched for information about drug use; 28% reported searching at least once per month. Participants generally (79%) preferred to obtain information online, but 62% also reported sharing and obtaining information about drugs in face-to-face interactions with friends and others. Concerns regarding the reliability of information, such as the presence of conflicting or contradictory messages from multiple sources, was a common theme in open ended responses. Of those who searched for information, about 70% reported desiring more detailed information about different drugs, in particular about their various side effects and risks. It is suggested that drug information campaigns, particularly those aimed at university students, might better focus on providing accurate, detailed information about the risks and other issues involved in various types of drug use, rather than one-size-fits-all messages. Given the varied and confusing nature of information already available, overly simplistic anti-drug messages may be ineffective, if not counterproductive.

## Introduction

Beginning with an initial pilot in 2003, Malaysia has gradually moved away from strictly punitive drug policies, centered around a mandatory death penalty for those convicted of trafficking, toward a more health-based model. The National Anti-Drug Agency (NADA) has successfully used harm-reduction measures such as needle exchange and methadone maintenance programs to curtail the spread of AIDS in addition to creating a network of cure-and-care facilities where individuals can obtain treatment voluntarily, free, and without legal implications (1). Nevertheless, in 2016, NADA reported the number of individuals entering or re-entering drug treatment had increased over the previous 5 years (2). This was also despite prevention and education campaigns utilizing various media that have reportedly been quite costly. Although there could be multiple explanations for such an increase (e.g., increased enforcement, higher levels of voluntary admissions), some have seen this as a reason to bring current prevention policies into question, even calling for a return to more draconian enforcement (3). Given that knowledge is a key part of prevention, and taking into consideration the results of a separate study where Malaysian youths reported a lack of reliable information (4), we took a brief look at the ways that Malaysians seek information about drug use and their reflections regarding the information that is currently available. The next section provides some background on information seeking related to drugs.

### Seeking information

Information seeking can take a variety of forms depending upon the motivations and circumstances of the individual (5, 6). Many American college students, for example, have been motivated to seek information about marijuana due to recent changes in its legal status (7). Watching previously banned substances become legal and even socially acceptable, cast doubt for many on the reliability of previously trusted information. Similar concerns also arose in a recent study of Malaysian university students (4).

Doubts about the reliability of information are common in relation to drug use. Stetina et al. for example, found that among those who had used recreational drugs, public health authorities and schools were seen as the least reliable sources of information (8): Users saw friends as trustworthy, while government sources were often seen as biased. Similarly, Lewis et al. reported that youths trusted government-provided information less than that provided by peers (7). Perceived trustworthiness may also be a function of the medium itself. Participants in several studies, for example, reported valuing social media as a means of promoting relationships, but not as a reliable source of information (9–11). In general, previous studies seem to indicate that although youths may be curious, and desire more information about drug use, they are not uncritical consumers of information. They appear to reconcile information received from sources such as government and media with that gathered from their own observations and experience. When they receive conflicting information they may be more likely to trust the experience of people they know well (i.e., peers) and their own observations (7).

To gain insight into how these processes work among Malaysians, this study examined the experiences of residents of four different regions of Malaysia related to searching for and sharing information about drugs.

## Methods

### Participants

This study used a convenience sample of participants recruited in public areas such as shopping centers and university campuses in four Malaysian states (Penang, Selangor, Malacca, and Johor). Any resident of Malaysia aged 18 or over was eligible to participate. The final sample included 280 participants (190 females and 90 males). Additional information about recruitment is included under Procedure. Additional demographic information is noted in Results and the Supplementary Files.

### Measures

Participants completed a 22-item paper-and-pencil questionnaire including both multiple choice and open-ended questions (Included in Supplementary Materials). Eighteen multiple choice items were taken from the Information Needs and Seeking Behaviour Questionnaire (12). For each of these questions, participants selected all applicable items from a list and also had the opportunity to respond in their own words in a space labeled “other.” Multiple choice questions assessed: the types of drug-related information searched for and shared; motivation for searching for and sharing drug-related information; preferred media for searching and sharing drug-related information; and perceived barriers to obtaining information related to drug use.

Participants also answered open-ended questions which asked (a) the types of media they used for obtaining information and why, (b) the information related to drug use that they were most interested in knowing, or felt was difficult to find, and (c) the kinds of information they shared about drug use and how they did so.

### Procedure

This study used a convenience sample. A booth was set up in areas with heavy foot traffic including university campuses, malls, and urban shopping areas from which participants were recruited through distribution of leaflets as well as snowballing/word of mouth. Each participant was paid RM 10 (about $2.40) to complete the survey. First, experimenters explained the purpose of the research, criteria for participation, and the right to withdraw at any time without penalty. Following this, participants signed the informed consent agreement and completed the survey. The questionnaire itself took between 15 and 30 min to complete. Participation, including explanation, consent, and debriefing, required about 30 to 40 min in total. A negligible number of participants (fewer than 5) withdrew after receiving the survey. To protect anonymity, no names or other identifying information were attached in any way to the questionnaire or responses. All materials and procedures were approved by the Monash University Human Research Ethics Committee (CF13/511–2013000227).

### Analysis

Quantitative data were analyzed using IBM SPSS 22. Qualitative data were organized and categorized using NVivo 11.

#### Coding

Open-ended responses were evaluated using thematic analysis. Coding techniques were modeled after those suggested by grounded theory (13). First, all open-ended text responses were reviewed, and a list of potentially important themes was generated. From this initial list, similar themes were grouped together into categorical codes. This procedure was conducted separately by two coders and the resulting categories compared to establish reliability. Overall agreement in coding assignments was 82.5%. Disagreements were resolved by the primary investigator.

## Results

### Demographic information

The final sample included 190 women and 90 men. Ages ranged from 18 to 65 (*M* = 28.23, *SD* = 11.347). Ethnic distribution was 46% Malay, 43% Malaysian Chinese, and 8% Malaysian Indian. Religious breakdown was 49% Muslim, 28% Buddhist, 11% Christian, 5% Hindu, and 5% Taoists. Educational distribution was 8% post-graduate, 37% university, 18% pre-university, 21% high school.

### Frequency of searching

Half of participants (*n* = 140, 50%), reported having searched for drug information at some time. Of these, 56 participants (20%) reported searching at least once per month, 13 (4.6%) searched at least once per week, and 9 (3%) reported searching for drug information at least once per day. (Table 1 in Supplementary Materials reports a breakdown of searching by region as well).

### Frequency of sharing

One hundred and thirteen participants (40%) reported sharing information on drug use. Fifty-seven participants (20.4%) reported sharing at least once per month, eight participants (2.9%) reported sharing at least once per day while seven participants (2.5%) reported sharing drug information at least once per week. In the empty space provided, participants reported sharing information under specific conditions such as: when drug use was discussed with peers or among social networks; when asked by a friend; when assigned to do so in school.

### Searching

#### Reasons for searching

The most common reasons given for searching out drug-related information were increasing awareness (*n* = 192, 69%), satisfying curiosity (*n* = 165, 59%), and concern for/helping loved ones (*n* = 111, 39.6%). Other reasons given include fulfilling academic requirements (*n* = 69, 24.6%), dissatisfaction with other information sources such as the school, workplace, and community-level prevention programmes (*n* = 54, 19.3%), and reducing uncertainty (*n* = 32, 11.4%). (Table 2 in Supplementary Materials summarizes this information).

Reasons for sharing drug information included increasing public awareness (n = 195, 69.6%), helping others (*n* = 124, 44.3%), social engagement (*n* = 97, 34.6%), a sense of civic duty (*n* = 96, 34.3%), and community interest (*n* = 88, 31.4%).

#### Preferred media for searching

Most participants preferred, in general, to search for information online (*n* = 223, 79.6%). Many (*n* = 120, 42.9%) also reported getting information from newspapers and magazines. Online/social media resources referenced frequently by participants were Google (*n* = 178, 63.6%), Facebook (*n* = 142, 50.7%), and YouTube (*n* = 120, 42.9%). (Table 3 in Supplementary Materials also summarizes this information).

#### Reasons for choosing specific media

Qualitative responses most often indicated an appreciation of the internet's convenience, accessibility, and low cost. Some also mentioned the ease of learning new information through videos, audio, slideshows, and other means obtainable online. Concerns about the reliability and accuracy of information obtained online was a common theme.

### Sharing information

#### Means of sharing

Face-to-face interactions were the most common way of sharing information (*n* = 170, 60.7%), followed by social media, specifically Facebook and WhatsApp (*n* = 157, 56.1%). A sizable portion of participants also indicated having shared drug-related information online through other means such as blogs and discussion forums (*n* = 92, 32.9%).

### Barriers to obtaining information

Of those who searched for information about drug use, 91 participants (65%) reported problems accessing the information that they desired. Unfamiliar language or jargon was a problem for 55 participants (39%); 42 participants (30%) reported doubts about the accuracy of information available; 35 participants (25%) reported being unable to find information relevant to their specific questions. Fourteen participants (10%) expressed fears or hesitation related to obtaining information, often related to privacy or anonymity.

### Types of information searched for, shared, and desired

#### Information searched for

The most common type of information sought related to the side-effects of specific drugs (*n* = 215,76.8%). Over half (*n* = 155, 55.4%) also searched for information on varieties of drugs. Information about drug symptoms was sought by 136 participants (48.6%). Other common search items were: drug prevention (*n* = 98, 35.0%), treatment approaches (*n* = 71, 25.4%), relapse prevention (*n* = 46, 16.4%), and rehab services (*n* = 43, 15.4%).

#### Types of information shared

The most common type of information shared related to the side effects of drug use (*n* = 212, 75.7%). Other types of information shared included methods of using/abusing drugs (*n* = 128, 45.7%), information about police actions/drug enforcement (*n* = 106, 37.9%), news about celebrities involved with drugs (*n* = 82, 29.3%), and news related to various designer drugs (*n* = 68, 24%).

### Desired information

Open-ended responses related to desired information fell into several broad categories which are outlined below.

#### Side-effect of drugs

About one-third (*n* = 98, 35%) of participants indicated that they desired more accurate or reliable information about the side-effects of drug us. Specifically, participants wanted more detailed information about overdosing and its causes, symptoms and effects with various drugs, both legal and illegal. Participants also expressed interest in whether some groups such as those suffering from specific health conditions or different ages and genders might be more at risk for negative side-effects.

#### Reliable information

One-out-of-eight participants (*n* = 35, 12.5%) discussed concerns about the credibility, or reliability, of available information. Again, many of these participants desired more specific information about the risks related to different drugs and medications. The information available, it was often reported, was either too general or too one-sided in its presentation.

#### New drugs and usage trends

Almost one-in-ten (*n* = 27) participants wanted accurate information on new drugs and trends in drug use. Questions related to the accessibility of new drugs, and a desire for more detailed information regarding new and designer drugs such as the risks and legalities involved. Others had questions regarding the use of new techniques for consumption, such as vaping, and the use of drugs in date rape.

#### Drug treatment

Eighteen participants (6.4%) expressed curiosity about drug treatment methods and their effectiveness.

## Discussion

Overall, one-half of respondents (140 out of 280) reported searching for information related to drugs at some point. Sixty-nine, about one-in-four, participants searched either weekly or monthly for drug-related information. Significantly, 91 participants (65% of those who searched) reported barriers or difficulties finding the information that they desired. Some reported problems understanding technical terms (14), but more often they expressed concerns about the reliability of the information available, or its' lack of desired details. All in all, about one-third of those surveyed here desired information about drugs or drug use, but had difficulty getting their questions answered. Who might best provide such information, and how best to do so are questions we hope to begin to address in this discussion.

A common motivation for information seeking was to answer, or find additional information about, questions that arose in conversation or appeared in various (often social) media (15). Although they reported using social media extensively, many participants reported being wary of the accuracy of information posted online (16, 9). About 30% of those who searched for information on drugs felt that the information available online was often biased in nature, with both individuals and government-sponsored organizations more interested in promoting opinions or specific agendas rather than presenting objective information (10). Others (about 39%) reported that, at times, information was too technical for them to understand.

Although participants were most likely to search for information online, most (61%) felt that information they received face-to-face was more reliable. Youths especially, expressed distrust for what they perceived as simplistic, one-sided messages often promoted by the government. For example, NADA officials continue to refer to the undifferentiated term “drugs” as Malaysia's “number one enemy” without much explanation or support in many communications (17). Studies from the US and the UK have also found simple authoritarian approaches to drug education to be largely ineffective (18). As in previous studies, respondents here were most interested in information received from individuals with experience or personal knowledge about drug-related risks (19).

The presence of conflicting information was an issue for many: i.e., what is “true” and what is just someone's agenda? The movement toward marijuana legalization in the US and Canada, in particular, has caused a kind of dissonance for many: Sometimes marijuana is portrayed as relatively benign. Other times, it is grouped together with arguably more addictive, and harmful, substances such as heroin and methamphetamine (20). In a similar vein, individuals may have acquaintances who have used marijuana or other substances with no apparent negative effects. However, they are told by others that if they experiment at all they could end up as addicts in the streets. In this light, it is unsurprising that, for many, *reliable* and *detailed* information was desired−70% of those who searched for information wanted to know about side-effects: They wanted details such as exactly how, and in what circumstances, these substances are potentially dangerous and, how various substances differ from each other in their effects and their risks.

## Limitations

The data presented here have numerous limitations. Most obviously, the sample was not representative of Malaysia in its makeup. This sample was 68% female compared to the general population which is about 48.3% female. This sample was also more heavily Malaysian Chinese in ethnicity (43% here vs. 23% population-wide) and included fewer Malays and Muslims than a representative sample would (about 62% of Malaysians are Muslim compared to 49% in this sample). Relatedly, these data were collected from a convenience sample via self-report: Participants probably self-selected to some degree and their responses likely include some self-presentation bias. It is not immediately clear how these factors might influence the trends seen here, nevertheless care should be taken not to overinterpret the results.

## Recommendations

When considered in the context of other research, these data, despite their limitations, can provide some perspective on these issues. Most notably, 35 percent of all respondents (70% of those who searched) reported desiring *more detailed* information about drug use and related issues such as their various side-effects. Relatedly, many cast doubt upon the reliability of the information that was available to them, particularly online. Given the global reach of the internet and the limited nature of these data we don't know exactly what information these participants were accessing. However, responses here as well as previous studies [e.g., (8, 11)] suggest that both social media and government-issued information are often seen as biased (this is likely true worldwide, not just in Malaysia). The responsibility for educating the public, thus, may best be fulfilled by concerned third-parties, such as NGOs, that work separately from government policies and law enforcement. If such groups can position themselves in a purely health- and well-being-oriented space where they are not specifically anti- or pro-drug, they are much more likely to be seen as unbiased, and therefore worthy of trust. In our view, the NZ Drug Foundation provides an excellent example of such an approach (21). The NZ Drug Foundation's website provides in-depth information on common illicit substances such as alcohol, marijuana, heroin, methamphetamine etc. in a clear, open fashion. Each substance's effects and side effects, signs of addiction and overdose, as well as when and how to seek help are explained clearly, in a non-judgmental fashion. Interested parties in Malaysia might benefit greatly from cooperating with and learning from such existing organizations.

A final point to keep in mind is that, given the complexity of the issues involved and the volumes of conflicting information available, aggressively pushing overly simplified messages may backfire. Simply grouping all “drugs” together as a “menace,” and all users as “addicts” (22) does not help curious individuals understand the nature of the problems involved. When faced with conflicting information, especially personal observations that contradict a view of drugs as the “number one enemy,” many might be tempted to distrust anything coming from such a source, no matter how well meaning they might be. Although a proportion of the population may be content to accept simple anti-drug messages at face value, when individuals opt to take the time to actively search for more information (as 50% of participants in this study had done), they are doing so because they desire more information: They are looking to make more informed decisions or help others do so, not to simply be told how to behave. Educators, and others wishing to promote public health in Malaysia, and elsewhere, should do their best to assist with this process. This can be done without encouraging drug use but, for many, it might also require becoming better informed personally about the issues involved.

## Author contributions

QC and CT designed the questionnaire and collected data. GB and QC interpreted the results and composed drafts of the manuscript. All authors participated in the data analysis and editing.

### Conflict of interest statement

The authors declare that the research was conducted in the absence of any commercial or financial relationships that could be construed as a potential conflict of interest.
