# Supplementary material for: Malaysians' Preferences and Concerns Regarding Seeking Information About Illegal Drugs
Source: Front Public Health. 2018 May 17;6:143. doi: 10.3389/fpubh.2018.00143 (PMC5966702; doi:10.3389/fpubh.2018.00143)
Supplement: Supplementary file 1 [file Table_1.DOCX]

**Supplemental Materials**

**Information Needs and Seeking Behaviour Questionnaire**

**Part I: Demographics**

**Instructions:** Please answer all questions below. For multiple-choice questions, please circle to indicate your response.

Q1. Age: _______________ years

Q2. Gender:

1. Male
2. Female

Q3. Nationality:

1. Malaysian
2. Others: (Please specify: _________________________)

Q4. Ethnicity:

1. Malay
2. Chinese
3. Indian
4. Others: (Please specify: _________________________)

Q5. Religion:

1. Muslim
2. Christian
3. Buddhist
4. Taoist
5. Hindu
6. Sikh
7. Others: (Please specify: _________________________)

Q6. Educational Status:

1. SPM/high school certificate
2. Pre-university programme
3. Diploma/equivalent
4. Bachelor degree
5. Postgraduate degree (Master/PhD)

**Part II: Information Seeking Behaviour and Medium**

**Instructions:** Please answer the items below where relevant. To indicate your response, please circle. For items Q9 and Q11, multiple responses are accepted where relevant.

Q7. Have you ever sought information on drug use?

1. Yes
2. No * (Go to Q9)

Q8. How frequently do you search for information on drug use?

1. At least once per day
2. At least once per week
3. At least once per month
4. Others: (Please specify: __________________________________________)

Q9. Please indicate the type of information you have searched/would search for. ***(You may circle more than one response)***

1. Drug types
2. Symptoms of drug misuse
3. Side-effects of drugs
4. Preventing drug misuse
5. Preventing drug relapse
6. Drug rehab treatment services
7. Types of treatment approaches
8. Others: (Please specify: _______________________________________________)

Q10. What motivated/would motivate you to search for information on drug misuse?

***(You may circle more than one response)***

1. Curiosity
2. Self-learning and increasing awareness
3. Seeking helpful information on behalf of a loved one/friend
4. Regain sense of control due to uncertainty
5. Academic purposes (e.g., to fulfil the requirement of an assignment)
6. Dissatisfied with the information received from other sources (e.g.: school, workplace, prevention programmes for the community)
7. Others: (Please specify: _______________________________________________)

Q11. Which medium have you used/would you use to search for information on drug misuse?

***(You may circle more than one response)***

1. Books
2. Newspaper/magazine articles
3. Brochures/pamphlets
4. Audio-visual teaching and learning materials
5. Internet websites/blogs/forums

(Please specify: __________________________________________)

1. Social media

Q12. Which social media sites have you used/would you use in your search?

1. Facebook
2. Twitter
3. Academia.edu
4. Google+
5. Tumblr
6. YouTube
7. Bebo
8. Others: (Please specify: __________________________________________________)

Q13. Why did you choose these medium to seek information?

1. Convenience (ease, availability and speed)
2. Privacy of information seeking experience
3. Credibility and reliability of information
4. Interactive methods of learning
5. Others:

Please specify below: ___________________________________________________________________________________________________________________________________________________________________________________________________________________________________________________________________________________________________________________________

Q14. What type of information about drug misuse would you like to see more of, on your preferred medium?

_______________________________________________________________________________________________________________________________________________________________________________________________________________________________________________________________

Q15. Have you encountered any barriers in searching for information related to drug misuse and prevention?

1. Yes
2. No * (Proceed to Part III)

Q16. What are the barriers that you have encountered?

1. Unfamiliar with language/jargon used
2. Unable to seek and obtain required information
3. Unsure of the credibility and reliability of information
4. Hesitant to approach a teacher/field expert/health professional
5. Others: (Please specify: _____________________________________________________)

**Part III: Information Sharing Behaviour and Medium**

**Instructions:** Please answer the items below where relevant. To indicate your response, please circle. For items Q19, Q21 and Q22, multiple responses are accepted where relevant.

Q17. Have you ever shared information related to drug misuse?

1. Yes
2. No * (Go to Q19)

Q18. When was the most recent occasion in which you shared information on drug misuse?

1. At least once per day
2. At least once per week
3. At least once per month
4. Others: (Please specify: __________________________________________)

Q19. Which medium did you use/would you use to share information? ***(You may circle more than one response)***

1. Face-to-face interaction with family and friends
2. Internet blogs/forums
3. Text/multimedia messaging (SMS/MMS)
4. Email
5. Social media
6. Others: (Please specify: _________________________________)

Q20. Why did you choose these medium to share information?

______________________________________________________________________________________________________________________________________________________________________________________________________________________________________________________________________________________________________________________________________________________

Q21. What type of information would you choose to share? ***(You may circle more than one response)***

1. Articles on latest drug raids by the police
2. Conventional and new designer drug types
3. New drug misuse methods (e.g., vaping)
4. Side-effects of drugs
5. Celebrities involved in drug misuse
6. Others: (Please specify: ______________________________________________________)

Q22. What motivated/would motivate you to share information about drug misuse? ***(You may circle more than one response)***

1. Sense of civic duty
2. Increase public awareness
3. Social engagement
4. Community interest
5. Enjoyment in helping others
6. Others: (Please specify: __________________________________________________)

Table 1.

*Crosstabulation of Geographical Location and Tendency to Search and Share Drug Information*

|  | Participants (N=280) | | | | Pearson’s Chi-Square | |
| --- | --- | --- | --- | --- | --- | --- |
| Information Searching Behaviour | Geographical Location | | | |  |  |
|  | Penang | Selangor | Malacca | Johor | χ^2^ (df=3) | p |
| Yes | 24 (49%) | 41 (80.4%) | 20 (31.3%) | 55 (47.4%) | 28.174 | .000** |
| No | 25 (51%) | 10 (19.6%) | 44 (68.8%) | 61 (52.6%) |  |  |
| Information Sharing Behaviour |  |  |  |  |  |  |
| Yes | 19 (38.8%) | 31 (60.8%) | 20 (31.3%) | 43 (37.1%) | 11.618 | .009** |
| No | 30 (61.2%) | 20 (39.2%) | 44 (68.8%) | 73 (62.9%) |  |  |

*Note:* **p<.01 (two-tailed)

Table 2.

*Factors Motivating Drug Information Searching and Sharing*

| **Motivating Factors** | **Responses (n, %)** |
| --- | --- |
| **Drug Information Searching** |  |
| Self-learning and increasing awareness | 192 (68.6%) |
| Curiosity | 165 (58.9%) |
| Seeking information on behalf of loved ones | 111 (39.6%) |
| Academic purpose (assignment) | 69 (24.6%) |
| Dissatisfied with other information sources | 54 (19.3%) |
| Regaining control due to uncertainty | 32 (11.4%) |
| Educating children and preventing drug misuse | 3 (1.07%) |
| Never searched | 1 (0.4%) |
| **Drug Information Sharing** |  |
| Increase public awareness | 195 (69.6%) |
| Enjoyment in helping others | 124 (44.3%) |
| Social engagement | 97 (34.6%) |
| Sense of civic duty | 96 (34.3%) |
| Community interest | 88 (31.4%) |
| No reasons provided | 2 (0.7%) |
| Self-education | 1 (0.4%) |
| Caring for the young | 1 (0.4%) |
| Creating a healthy community | 1 (0.4%) |
| No specific intention | 1 (0.4%) |

Table 3.

*Preferred Media to Search and Share Drug Information*

| **Preferred Medium** | **Responses (n, %)** |
| --- | --- |
| **Drug Information Searching** |  |
| Websites/blogs/forums | 223 (79.6%) |
| Newspapers and magazines | 120 (42.9%) |
| Social media | 84 (30.0%) |
| Brochures and pamphlets | 68 (24.3%) |
| Books | 58 (20.7%) |
| Audiovisual materials | 29 (10.0%) |
| **Drug Information Sharing** |  |
| Face-to-face interaction | 170 (60.7%) |
| Social media | 157 (56.1%) |
| Internet website/blog/forum | 92 (32.9%) |
| SMS/MMS | 57 (20.0%) |
| Email | 20 (7.1%) |
| School education and events | 4 (1.4%) |
